# The approach to hip instability in children with cerebral palsy: an umbrella review

**DOI:** 10.1530/EOR-2025-0114

**Published:** 2026-03-02

**Authors:** Ana Paula Tedesco, Alessandro Melanda, Davi Moshe, Epitácio Rolim Filho, Francesco Camara Blumetti, Leonardo Cury Abrahão, Mauro César de Morais Filho, Patricia Moreno Grangeiro

**Affiliations:** ^1^ Instituto de Neuro-Ortopedia, Caxias do Sul, RS, Brazil; ^2^ Universidade Estadual de Londrina – Paraná, Instituto Pró-Kids de Londrina, Londrina, PR, Brazil; ^3^ Universidade Federal do Ceará, Fortaleza, CE, Brazil; ^4^ Departamento de Ortopedia da UFPE, Hospital Getúlio Vargas, AACD, Recife, PE, Brazil; ^5^ Hospital Israelita Albert Einstein, São Paulo, SP, Brazil; ^6^ Ortopédico BH; AMR; Hospital da Baleia, Belo Horizonte, MG, Brazil; ^7^ AACD – Associação de Assistência à Criança Deficiente, São Paulo, SP, Brazil; ^8^ Centro Integrado de Neuro-Ortopedia, Instituto de Ortopedia e Traumatologia, HCFMUSP, Universidade de São Paulo, São Paulo, SP, Brazil

**Keywords:** cerebral palsy, guidelines, hip instability, surveillance, umbrella review

## Abstract

**Purpose:**

- Children with cerebral palsy (CP) are at a high risk of progressive hip displacement, defined as lateral migration of the femoral head measured by the Reimers migration percentage. This condition may impair quality of life, highlighting the need for improved hip care. This umbrella review assessed current evidence on the evaluation, prevention, and treatment of hip displacement in children with CP by synthesizing systematic reviews.

**Methods:**

- Systematic reviews published in English between 2004 and 2024 were included, focusing on children with CP aged 0–18 years. Searches were conducted in nine databases: PubMed, MEDLINE, Web of Science, Scopus, BVS, CINAHL, Cochrane Library, PEDro, LILACS, and EMBASE. Methodological quality was assessed using AMSTAR 2 for interventional studies and JBI criteria for non-interventional studies. This review has been registered at PROSPERO (registration number: CRD42024618645).

**Results:**

- In total, 25 systematic reviews addressed key aspects of hip management, including hip surveillance; tone management; preventive, reconstructive, and salvage procedures; and antifibrinolytic use. Hip surveillance reduced hip dislocation rates and the need for salvage surgery, whereas postural and tone management showed no consistent preventive effect. Combined pelvic and femoral osteotomies achieved better outcomes than isolated procedures, while soft-tissue surgeries had high recurrence rates. Proximal femoral hemiepiphysiodesis improved radiographic outcomes but frequently required revision. Salvage procedures such as valgus osteotomy and femoral head resection relieved pain, whereas hip arthrodesis showed poor outcomes. Total hip arthroplasty improved pain and function but was associated with high complication rates.

**Conclusions:**

- This umbrella review highlights evidence-based practices and important knowledge gaps in the management of hip instability in children with CP, supporting future research and improved clinical care.

## Introduction

Hip instability in children with cerebral palsy (CP) is a multifactorial condition caused by muscular imbalance, abnormal tone, and bony deformities that predispose the hip to loss of femoral head containment. This pathophysiological state often progresses to hip displacement, defined as the lateral migration of the femoral head from the acetabulum. Displacement is objectively assessed by the migration percentage (MP), as described by Reimers (1), which is central to hip surveillance (HS) and clinical decision-making in CP (2). Progressive displacement predisposes the hip to subluxation or dislocation and, if left unaddressed, may lead to pain, impaired sitting balance, difficulty with ambulation, and reduced quality of life (QoL) (3). Definitions of hip displacement vary widely, with MP thresholds ranging from 10 to 40% for hips considered at risk, 30–59% for subluxation, and ≥90–100% for dislocation (4, 5, 6).

Hip instability in CP patients is also strongly associated with gross motor function, as classified by the Gross Motor Function Classification System (GMFCS) (7). The prevalence of hip displacement is approximately 35% overall, ranging from 0% at GMFCS level I to 90% at GMFCS level V (7). Regular radiographic assessments and standardized screening protocols are essential for effectively addressing hip instability (8, 9). Management options, including physiotherapy, botulinum toxin A (BoNTA) injections, neurosurgical approaches such as intrathecal baclofen (ITB) pumps, and selective dorsal rhizotomy (SDR), are described in the literature (10). Orthopedic surgeries – including soft-tissue and bone procedures such as proximal femoral guided growth and femoral and pelvic osteotomies – and salvage procedures – including total hip replacement – have been described in several studies (11, 12). These studies frequently report complications, such as recurrence, residual femoral and acetabular deformities, avascular necrosis, blood loss, and prolonged hospital stays.

Establishing evidence-based guidelines for monitoring, diagnosing, and treating hip instability has shown measurable benefits, including improved clinical outcomes (9, 13). Despite sufficient scientific evidence supporting the development of hip screening programs in CP, there is a lack of high-level evidence to support any specific management algorithm for the prevention or treatment of hip displacement in this population (14).

This study seeks to consolidate the current knowledge on diagnosis, surveillance protocols, and interventions for the prevention and treatment of hip instability in patients with CP. Using an umbrella review methodology, we aim to evaluate existing evidence from published systematic reviews, identify inconsistencies, and highlight areas requiring further research, ultimately enhancing clinical outcomes and QoL. Although surgical decision-making in hip instability for children with CP is inseparable from the surgeon’s experience and the family’s willingness, particularly when the child is asymptomatic, we aimed to complement this context with objective, evidence-based factors to support individualized, shared decision-making.

## Methods

A preliminary search of MEDLINE, Cochrane Database of Systematic Reviews, and JBI Evidence Synthesis was conducted, and no current or ongoing reviews of systematic reviews on the topic were identified. An unpublished protocol was drafted and is available upon request by the author. This review was registered in the PROSPERO database (CRD42024618645).

### Selection criteria

This umbrella review included systematic reviews of children with CP (0–18 years old). Studies published in English and Portuguese between 2004 and 2024 were included.

### Search strategy

An initial limited search of PUBMED/MEDLINE was performed to identify articles on the topic. The text words contained in the titles and abstracts of relevant articles and the index terms used to describe the articles were selected to develop a complete search strategy for peer-reviewed journals on CINAHL, Biblioteca Virtual em Saúde (BVS), Web of Science, Scopus, Physiotherapy Evidence Database (PEDro), and EMBASE (see Appendix 1 (see section on Supplementary materials given at the end of the article)). The search strategy, including all the identified keywords and index terms, was adapted for each database. The reference list of all included sources of evidence was screened for additional studies.

### Study selection

Following the search, all identified citations were uploaded to Rayyan (https://rayyan.qcri.org) (15) and duplicate entries were automatically removed. Two reviewers independently evaluated the titles and abstracts to assess their adherence to the review’s inclusion criteria. Any disagreement between the reviewers at each stage of the selection process was resolved through discussion with the other authors. The results of the search and study inclusion process were fully reported in the final review in a Preferred Reporting Items for Systematic Reviews and Meta-analyses (PRISMA) flow diagram (16). The PRISMA 2020 checklist is available as a supplementary file.

### Assessment of methodological quality

Full-text articles were retrieved, and the authors performed a critical appraisal using A MeaSurement Tool to Assess Systematic Reviews (AMSTAR 2) (17) to evaluate the methodological quality of systematic reviews of intervention studies. For non-intervention studies, the Joanna Briggs Institute (JBI) critical appraisal tool was used to assess methodological quality and risk of bias (18).

### Data extraction and outcomes

Data were organized into categories determined by the aim of this umbrella review. The data extracted were synthesized in tables containing study variables including authors, year of publication, title of the study, type of study, the number of reviewed studies, level of evidence, population, GMFCS level distribution, age at intervention and follow-up, results, complications, and authors’ conclusions.

## Results

### Selection of included reviews

**Figure 1:**
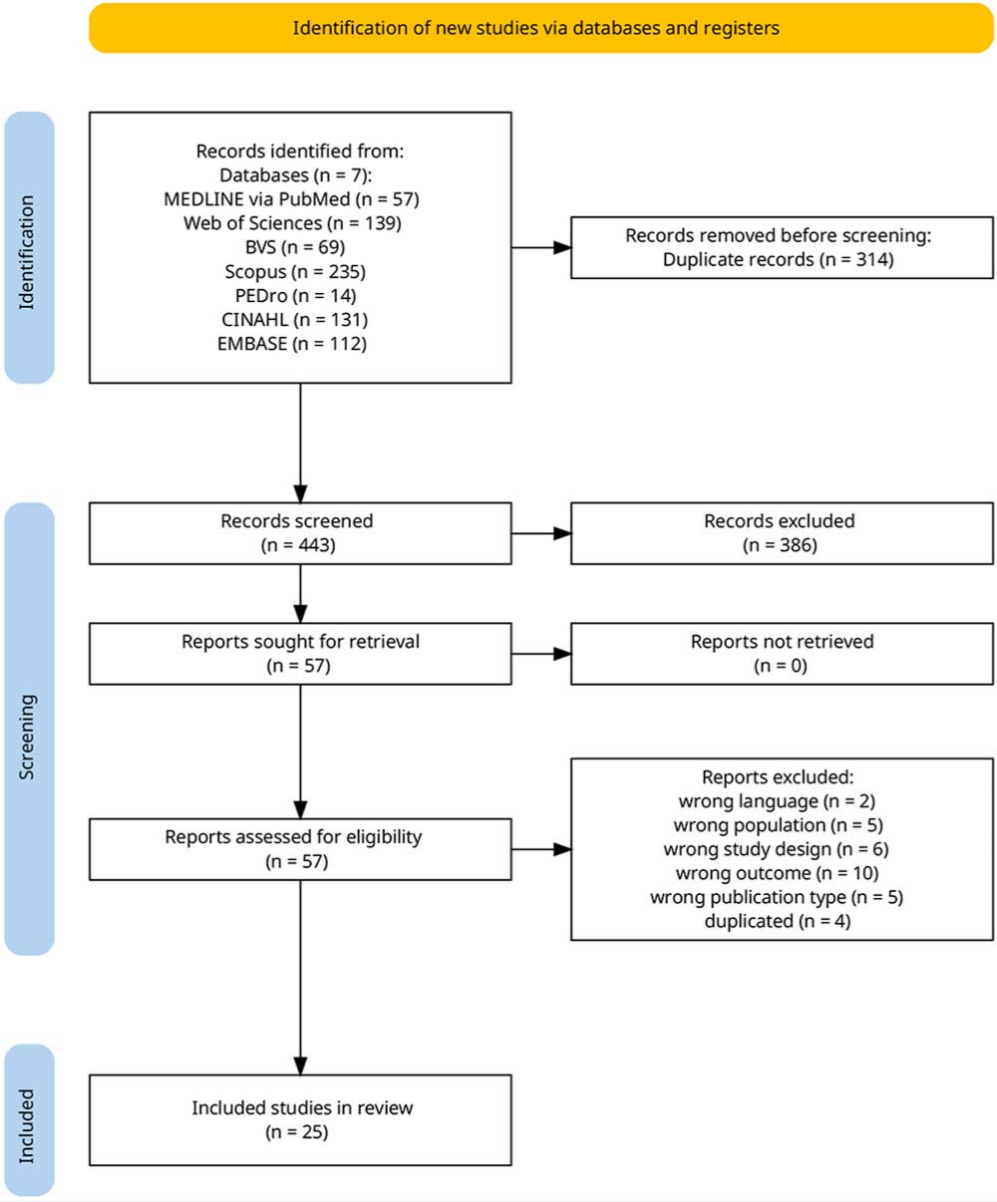
PRISMA 2020 flow diagram. The selection process of systematic reviews included in this umbrella review. Of the 754 records identified across nine databases, 25 met the inclusion criteria. Reasons for exclusion are provided in the supplementary material.

A total of 754 studies were identified across seven databases (Fig. 1). After removing duplicates (*n* = 314), 443 studies underwent title and abstract screening and 57 proceeded to full-text review. Finally, 25 articles were identified as eligible. The excluded articles (and the reasons for exclusion) are provided (Supplementary material).

### Quality appraisal of selected reviews

Table 1 presents the results from the critical appraisal checklist used to guide the comparison of low- and high-quality reviews and the potential impact on the interpretation of results from this umbrella review, according to AMSTAR 2 (17). The JBI tool scores are shown in Table 2.

**Table 1:** Assessment of the methodological quality of the systematic reviews included, using the AMSTAR 2 instrument.

Studies	1	2	3	4	5	6	7	8	9	10	11	12	13	14	15	16	Ratio of yes	Criteria
De Dios Domínguez *et al.* (42)	Y	N	Y	Y	Y	PY	N	Y	N	N	N	N	N	N	N	Y	15/16	Critically low
De Souza *et al.* (34)	Y	N	Y	Y	PY	PY	N	Y	N	N	N	N	N	N	N	Y	13/16	Critically low
Rangasamy *et al.* (41)	Y	Y	Y	Y	Y	Y	PY	Y	Y	N	Y	Y	Y	Y	Y	Y	15/16	High
Costa *et al.* (37)	Y	N	Y	Y	Y	Y	N	Y	PY	N	N	N	N	N	N	Y	16/16	Critically low
Miller *et al.* (10)	Y	Y	Y	Y	Y	Y	Y	N	Y	N	Y	Y	N	N	Y	Y	12/16	Low
Agarwal *et al.* (33)	Y	Y	Y	Y	Y	Y	Y	Y	Y	Y	Y	Y	Y	Y	Y	Y	16/16	High
Meyling *et al.* (21)	Y	N	N	PY	Y	Y	N/A	PY	N	N	N/A	N/A	N	Y	N	N	4/16	Critically low
Hesketh *et al.* (40)	Y	Y	Y	Y	Y	Y	Y	Y	Y	N	Y	Y	Y	Y	Y	N	14/16	Critically low
Kolman *et al.* (35)	Y	Y	Y	Y	Y	Y	Y	Y	Y	Y	N	Y	Y	Y	Y	Y	13/16	Low
Boldingh *et al.* (36)	Y	N	Y	Y	Y	Y	Y	Y	Y	Y	N	N	Y	Y	N	Y	13/16	Critically low
Lebe *et al.* (28)	N	Y	Y	Y	Y	Y	Y	Y	Y	N	Y	Y	Y	Y	Y	Y	14/16	Moderate
Larrague *et al.* (38)	Y	N	Y	Y	Y	Y	Y	Y	Y	N	N	PY	N	Y	Y	Y	15/16	Low
Pin *et al.* (25)	N	N	Y	PY	Y	N	N/A	N	N	N	N	N	N	N	N	N	2/16	Critically low
Bouwhuis *et al.* (26)	Y	Y	Y	Y	Y	Y	Y	Y	N	N	Y	N	N	Y	Y	Y	12/16	Critically low
Adams & Lakra (39)	Y	N	Y	Y	Y	Y	N	Y	N	N	N	N	N	N	N	Y	13/16	Critically low
El-Sobky *et al.* (32)	Y	Y	Y	Y	Y	Y	PY	Y	Y	N	Y	Y	Y	Y	N	Y	14/16	Low
Blake *et al.* (20)	Y	Y	Y	Y	Y	Y	PY	Y	Y	Y	N/A	N/A	Y	Y	N/A	Y	12/16	High
Pin (22)	N	N	Y	Y	Y	N	Y	N	N	N	N	N	N	N	N	N	4/16	Critically low
Davidson *et al.* (24)	Y	Y	Y	Y	Y	Y	Y	Y	N	N	Y	N	N	Y	Y	Y	12/16	Critically low
Perez-de la Cruz (23)	Y	Y	Y	Y	N	Y	N	Y	N	N	Y	N	N	N	N	Y	8/16	Critically low

Y, yes; N, no; PY, partially yes; and N/A, not applicable.

**Table 2:** Critical appraisal of the systematic reviews of non-intervention studies included, using the Joanna Briggs Institute (JBI) instrument.

Studies	1	2	3	4	5	6	7	8	9	10	11	Ratio of yes	Criteria
Karkeny *et al.* (3)	Y	Y	Y	Y	Y	Y	Y	Y	Y	Y	Y	11/11	Excellent
Pons *et al.* (19)	Y	Y	Y	Y	Y	Y	Y	Y	N	Y	Y	10/11	Excellent
Barik *et al.* (2)	Y	Y	Y	Y	N	U	Y	Y	N	Y	Y	8/11	Excellent
Gordon & Simkiss (8)	Y	Y	Y	Y	Y	Y	U	Y	N/A	Y	N	8/11	Excellent
Wynter *et al.* (9)	Y	Y	Y	Y	Y	Y	Y	Y	N/A	Y	Y	10/11	Excellent

Y, yes; N, no; U, unclear; and N/A, not applicable.

## Outcomes

### Pain

One study was selected on the subject. Karkeny *et al.* (3) conducted a systematic review to determine whether pain is independently associated with hip migration in patients with severe CP. The review identified 15 articles that demonstrated this association. However, only five studies (four were classified as level III evidence and one level II) provided robust evidence according to the authors’ quality criteria, which included the presence of a control group without pain, a minimum of 50% of participants classified as GMFCS IV and V, assessment of pain quality and laterality, and the use of Reimers’ MP for hip evaluation. Significant limitations identified in these five studies included a lack of standardized methodology for classifying hip dysplasia and for assessing and describing pain, as well as age variation among participants (9.5–40 years). The findings indicated that two studies did not support the pain–hip dysplasia correlation, two supported it (one identified MP > 50% as an independent pain risk factor and the other linked increased pain scores to Melbourne Cerebral Palsy Hip Classification Scale (MCPHCS)), and one study was inconclusive (MP in painful hips was 26% compared to 21% in painless hips).

### Radiological evaluations

Clinical decisions about interventions in children with CP depend heavily on radiological parameters assessing hip migration and geometry. Two high-quality systematic reviews by Pons *et al.* (19) and Barik *et al.* (2) evaluated the validity and reliability of these imaging methods.

Pons *et al.* (19) analyzed 19 studies that assessed the metrological properties of different radiographic measurements to evaluate their accuracy. Three-dimensional computed tomography (CT) was included in the comparative analysis but was not the sole focus of the investigation. The authors found excellent correlations of CT with neck–shaft angle (NSA) and MP, and with the Robin–Graham classification, and good correlations with AI and femoral anteversion.

They examined hip migration, acetabular dysplasia, NSA, head–shaft angle (HSA), and femoral anteversion using the Robin–Graham classification. Radiographs showed excellent reliability and validity for NSA and hip migration. The AI had good reliability but moderate validity. Femoral anteversion measurements correlated well between 2D CT and clinical assessment. The Robin–Graham classification demonstrated excellent intra- and inter-observer reliability and a strong correlation with 3D CT in children aged 2–7 years.

Barik *et al.* (2) included 30 studies with 5,560 CP patients (mean age: 8.5 years), 82.6% of whom were at GMFCS levels IV and V. The MP increased by 11% annually in quadriplegic patients, 2% in diplegic patients, and 0.5% in hemiplegic patients. MP progression was categorized as stable, increasing, or decreasing, with annual increases of 0.3, 1.9, and 6.2% in GMFCS levels I–III, IV, and V, respectively. An HSA > 164.5° was associated with MP > 40%, while an acetabular index >34° indicated severe dysplasia. Lateral center–edge angle (CEA) decreased with hip lateralization, and pelvic obliquity >5° correlated with future hip dysplasia and scoliosis. The CT femoral anteversion >46° was associated with increased MP and hip dysplasia.

### Hip surveillance

We identified two systematic reviews assessing the effectiveness of HS in CP (8, 9). Details of these studies are depicted in Table 3. Both studies were considered high quality based on the JBI scores. The HS was found to be effective in reducing the incidence of complete hip dislocation in CP, reducing the likelihood of reconstructive and salvage surgery, while increasing the indication for preventive surgery, such as soft-tissue release and proximal femoral hemiepiphysiodesis (PFHE) in early stages of displacement.

**Table 3:** Hip surveillance studies.

Study	Studies reviewed, *n*	Study type	Evidence	General descriptions/remarks	Results/author’s conclusions
Gordon & Simkiss (8)	6	All observational studies (due to the nature of the study)	High quality: 2; medium quality: 3; low quality: 1	Hip surveillance resulted in: i) Increase in preventive surgeries from 51 to 70.9% ii) Decrease in reconstructive surgeries from 37.1 to 29% iii) Reduction in the average age for preventive hip surgery from 8.3 to 4.2 y iv) Eliminated the need for salvage surgeries for dislocated hips in two studies	Children with bilateral CP should get a pelvic X-ray at 30 months, or earlier if clinical suspicion. Children with MP > 33%, or AI >30°, will generally require surgical treatment. Progression of MP > 7% per year requires close monitoring
Wynter *et al.* (9)	9	All observational studies (due to the nature of the study)	Level III: 8; level IV: 7; MINORS score: 5–14	Hip surveillance resulted in a significant decrease in the incidence of hip dislocation. Dislocation rates in monitored patients ranged from 0 to 6.9%. Cases of dislocation in monitored patients happened in the following situations: i) Dislocation is present in the initial radiograph, even in children as young as 3 years old ii) Dislocation happened after orthopedic referral, while waiting for surgery	Hip surveillance should start as early as 12–24 mo in GMFCS levels IV and V and should be less frequent for GMFCS I and II. Children with GMFCS I and hemiplegic CP, and a type IV Winters, Gage, and Hicks gait pattern, need closer monitoring. Surveillance after skeletal maturity in non-ambulatory patients and if risk factors are present (pelvic obliquity and scoliosis)

CP, cerebral palsy; MP, migration percentage; AI, acetabular index; mo, months; y, years; and GMFCS, Gross Motor Function Classification System.

### Postural and tone management

Postural and tone management strategies for hip displacement prevention in children with CP include positioning, bracing, and spasticity control via BoNTA, SDR, ITB, and nerve blocks. Five systematic reviews addressed positioning systems, bracing, and weight-bearing and are summarized in Table 4. Blake *et al.* (20) found no randomized trials on sleep positioning; two small studies showed no significant benefits. Miller *et al.* (10) found limited support for daily standing in hip abduction and modest short-term benefits from combining BoNTA with the Sitting, Walking, and Standing Hip (SWASH) orthosis brace. Meyling *et al.* (21) reported potential effects from standing frames and abduction supports, but small, heterogeneous samples weakened evidence. Pin (22) found moderate support for static weight-bearing improving bone density, but not for preventing hip displacement. Pérez-de la Cruz (23) found non-significant reductions in hip dislocation and surgery with orthotics and postural systems, often deemed uncomfortable. All reviews emphasized the lack of robust trials.

**Table 4:** Postural management studies.

Study	Studies, *n*		General description/remarks	Results/author’s conclusions
	Total	LOE		
Blake *et al.* (20)	0	Level 1 (Cochrane)	Compare commercially available sleep positioning systems to usual care in reduction or prevention of hip migration. No eligible trials found; highlighted the research gap on nighttime positioning systems	No evidence to confirm or refute effectiveness
Meyling *et al.* (21)	8	Level 3: 3; level 4: 4; level 5: 1	Inclusion and exclusion criteria were poorly described, particularly regarding GMFCS levels and age; studies focused on GMFCS III to V. Age at intervention start varied widely (5 mo to 18 y), and only two studies included children under 18 mo. Intervention details, including positioning (standing, sitting, lying, or combination), hip abduction angles, duration, and intensity, were inconsistently reported; no studies addressed adherence to the intervention	Limited evidence for postural management to prevent or reduce hip migration due to a lack of high-quality studies. There is a positive trend in using hip abduction in postural management. Strong clinical recommendations cannot be made based on the current evidence
Miller *et al.* (10)	8	Level 3: 2; level 4: 5; level 5: 1	Daily use and duration of intervention varied; different positioning devices included (standing devices, custom seating, 24 h postural management, and sleep systems) – six studies focusing on positioning hips in abduction; no studies discussed hip flexion/extension or internal/external rotation. Small samples, short follow-up (1–4 y)	Changes in MP reported in four studies (annual rates from −8% to +3.2%). Level III studies (2) suggested that standing with hips in abduction for more than one hour daily may positively impact hip displacement in GMFCS levels III to V. Level IV studies (5) found little or no effect, with one study reporting a negative association between standing time (hips in a neutral position) and hip displacement. Studies do not provide sufficient or consistent data to support specific interventions
Pérez-de la Cruz (23)	18	Level 2	Effectiveness of static weight-bearing – static and dynamic. Some studies also with BoNTA. Demographic aspects of the patients, methods, and results of revised studies not provided. Not possible to identify the data that led to the given conclusions	Although the literature does not support the use of postural systems in the treatment of hip deformities, authors found that these devices can help to control them. However, these systems must be used for prolonged periods of time before the effects can be observed
Pin *et al.* (22)	10	Level I: 6; level II: 1; level IV: 3	Compare commercially available sleep positioning systems to usual care in reduction or prevention of hip migration	Conclusion: no RCT was found that evaluated the effectiveness on preventing hip migration and the number or frequency of hip problems

LOE, level of evidence; GMFCS, Gross Motor Classification System; mo, months; y, years; and MP, migration percentage.

Three studies addressed tone management (Table 5). Davidson *et al.* (24) compared SDR and ITB in non-ambulatory children (GMFCS IV/V), finding that both reduced spasticity but without a significant impact on hip subluxation or need for surgical containment. Pin *et al.* (25) found BoNTA effective for post-surgical pain but not for preventing hip displacement. Miller *et al.* (10) reported limited evidence for non-surgical tone management methods, all of which were constrained by a poor study design and short follow-up.

**Table 5:** Tone management studies.

Study	Studies, *n*		General description/remarks	Results/author’s conclusions
	Total	LOE		
SDR/ITB				
Miller *et al.* (10)	4			Due to a lack of rigorously designed studies, current evidence is insufficient to support or refute IBP/SDR in the prevention of hip dislocation
IBP		Level 4: 1	Follow-up only one year; age: 4–31 y; all GMFCS levels/no exclusion of previous orthopedic hip surgery	Annual rate of change in MP: +1.3%, which the authors considered a positive result compared to the typical progression of hip displacement reported in the literature (+5% per year)
SDR		Level 4/5: 7	Percentage of afferent rootlets cut varied widely (5–85%)	Four studies may positively influence; one study reported an annual migration rate of +1.7%; three studies reported stability rates (82–93% of hips no worsening or improvement with a follow-up of 4 y or less). One study reported rapid progression of hip displacement in 6 patients, preexistent hip dysplasia was a predisposing factor, and hips with an intermediate degree of preoperative lateral extrusion (12–25%) had variable results
Davidson *et al.* (24)	27	Level 4	Studies most small and heterogeneous population; only four studies mentioning hip status, with no detailed information about demographic of patients, methods of evaluation or follow-up provided	Neither SDR nor IBP have a significant effect in hip subluxation nor in the need of hip containment procedures
BoNTA				
Pin *et al.* (25)	19*	Level 4	Mixed spastic (*n* = 26) and dyskinetic CP (*n* = 1), all GMFCS. Conclusion based on the only study level IV evidence	Moderate evidence – mean changes in MP of no clinical significance (<10%) over a 2-y follow-up
Miller *et al.* (10)	35 (mixed)	Level 3: 1; level 4: 3	Varied participant characteristics and injection protocols; single and repeat injections	One study showed a significant reduction in migration rates. One RCT combined BoNTA with SWASH bracing and reported a lower annual progression of MP and fewer surgeries in the intervention group, but long-term follow-up showed no sustained benefits. The evidence does not support recommending BoNTA for the prevention or slowing of hip displacement
Obturator nerve block				
Miller *et al.* (10)	1	Level 3	GMFCS levels III to V, initial MP 20%–60% compared with control group	Annual change MP: +1.09%. Obturator nerve block might be effective in controlling hip displacement in the short term; however, due to the low LOE, no recommendations can be made

* One study about hip results.

No, number; y, years; CP, cerebral palsy; BoNTA, botulinum toxin A; LOE, level of evidence; MP, migration percentage; SWASH, Sitting, Walking, and Standing Hip; GMFCS, Gross Motor Function Classification System; ITB, intrathecal baclofen; IBP, intrathecal baclofen pump; and SDR, selective dorsal rhizotomy.

### Orthopedic surgery

Surgical treatments in CP can be categorized into preventive, reconstructive, and salvage procedures. A total of five reviews (26, 28, 32, 33) evaluated interventions such as soft-tissue releases, PFHE, and hip reconstructive procedures, including femoral and pelvic osteotomies (Supplementary Table 1). Three studies (34, 35, 36) focused on salvage surgeries (Supplementary Table 2), while another three (37, 38, 39) specifically analyzed total hip arthroplasty (THA) (Supplementary Table 3). Additional aspects addressed in the literature included complications such as avascular necrosis (AVN) after hip reconstructive surgery and the role of antifibrinolytic agents, particularly tranexamic acid (TXA) and ε-aminocaproic acid (EACA) (Table 6).

**Table 6:** Antifibrinolytics studies.

Study	Studies/LOE			Population	GMFCS	Age at surgery	Results/complications	Authors’ conclusion
	Total	LOEI	LOEIII					
De Dios Domínguez *et al.* (42)	7	1	6	943 pts	NR	Under 18 y	Reduction in overall transfusion rate, postoperative transfusion rate, and TBL; there was a shorter length of stay in the control group (*P* = 0.01). No significant differences in EBL, intraoperative transfusion rate, postoperative Hb, postoperative Hct, drop in Hct, or drop in Hb. No adverse events or complications	TXA effectively reduces blood loss and decreases the need for total and postoperative transfusions
Rangazamy *et al.* (41)	5	1	4	478 pts	I (1), II (45), III (63), IV (182), V (129)	Mean 6.54–10.2 y	Three studies: antifibrinolytics (TXA or EACA) reduction in total blood loss with a mean difference of 151.05 mL (*P* = 0.01). Two studies: reduction EBL (statistically not significant). No adverse effects	Although two out of five included studies supported the use of antifibrinolytics, the evidence is not sufficient for a definitive conclusion to support its routine use

pts, patients; y, years; LOE, level of evidence; NR, not reported; TXA, tranexamic acid; EACA, ε-aminocaproic acid; EBL, estimated blood loss; TBL, total blood loss; Hb, hemoglobin; and Hct, hematocrit.

### Soft-tissue surgeries

Bouwhuis *et al.* (26) reviewed 15 studies evaluating the effectiveness of preventive and corrective surgical interventions for hip instability, five of which focused on soft-tissue surgeries. However, only one study met their quality criteria (27). This study analyzed 70 hips in 38 patients who underwent bilateral soft-tissue hip surgeries, transfer of adductor longus and the gracilis to the ischium, and psoas tenotomy (performed at the level of the lesser trochanter when flexion contracture was greater than 30°). Applying a strict follow-up criterion (MP ≤ 15%), 17% of the hips required additional surgery, with a mean follow-up period of 7.2 years.

### Proximal femoral hemiepiphysiodesis

Lebe *et al.* (28) conducted a meta-analysis showing that PFHE improves radiographic outcomes, including MP, HSA, and AI, with a follow-up of at least 2 years. Screw sizes varied according to neck diameter, ranging from 4.5 to 7.0 mm, and were partially or fully threaded, with at least three threads crossing the physis (29, 30). Hsieh *et al.* (31) recommended that the screw tip be placed in the medial third of the capital epiphysis on the anteroposterior view. Physeal growth off the screw is frequent (44%) and often requires revision (31).

### Reconstructive surgeries

El-Sobky *et al.* (32) reviewed 36 studies (1,771 patients) and concluded that femoral and acetabular osteotomies with soft-tissue release effectively reduce MP. Soft-tissue procedures focused on adductor release, with occasional iliopsoas tenotomy or other releases. Some studies assessed contralateral surgery to preserve symmetry. Comparative retrospective studies generally favored combined pelvic and femoral osteotomies over isolated varus derotation osteotomy (VDRO), particularly due to lower rates of resubluxation, dislocation, and related complications. Twelve retrospective studies with a long-term follow-up (over 6 years) evaluated outcomes of either combined pelvic and femoral interventions or isolated VDRO. Short- and long-term results also favored the combined approach, demonstrating satisfactory radiological and clinical outcomes. In contrast, VDRO-only studies often reported high recurrence rates, limited radiological and clinical improvement, and, in one study, a high rate of early reoperation on the contralateral hip.

Agarwal *et al.* (33) also showed that the combined pelvic and femoral osteotomy approach provided greater long-term hip stability. Among the 323 hips, 13.9% required reoperation, compared to 20.8% of the 303 hips treated with femoral osteotomy alone. The comparison between femoral osteotomy and soft-tissue surgery showed that 56.8% of hips treated with soft-tissue surgery alone required reoperation, whereas only 22.9% of those treated with femoral osteotomy required reoperation.

Bouwhuis *et al.* (26) reported postoperative hip pain outcomes from eight studies, but only five provided preoperative data. Among 93 patients, 81% had preoperative pain, but only 5% had pain at follow-up. However, the caregiver often reported pain without validated scoring. El-Sobky *et al.* (32) emphasized that most studies lacked standardized pain evaluation tools.

Complications were reported in all preventive and reconstructive studies, with rates up to 25% (26), including pain, AVN, fractures, reoperation or revision, redislocation, coxa vara, graft dislocation, osteoarthritis, infection, hardware failure, windblown deformity, sitting problems, deterioration of motor functions, heterotopic ossification (HO), recurrent contraction, pathological fracture and decubitus ulcer (26, 32).

### Salvage procedures

We have found systematic reviews on surgical procedures performed for pain relief, comfort, and ease of care when reconstructive surgeries are no longer possible or failed, including femoral osteotomies, hip arthrodesis (HA), and THA.

### Femoral osteotomies

Three studies (de Souza *et al.* (34), Kolman *et al.* (35), and Boldingh *et al.* (36)) evaluated femoral osteotomies as salvage procedures mainly in non-ambulatory individuals (GMFCS IV and V) presented with chronic hip pain or failed prior reconstructions. The main techniques assessed were proximal femoral valgus osteotomies (e.g., Schanz and McHale) and femoral head resection (FHR). Pain relief was reported in up to 93.3% of cases in Kolman *et al.* (35) and over 80% in de Souza *et al.* (34). A comparative analysis by de Souza *et al.* (34) found similar clinical outcomes between McHale osteotomy and FHR. Complications were more frequently associated with FHR, particularly HO (3.2–62%) and proximal femoral migration (7.7–28%), although at lower rates than those reported for THA.

### Hip arthrodesis

Hip arthrodesis was also evaluated in the reviews by de Souza *et al.* (34), Kolman *et al.* (35), and Boldingh *et al.* (36), which reported predominantly unfavorable outcomes regarding the procedure’s clinical efficacy and safety. Complications were reported in 17 patients in 16 procedures (non-ambulatory), including a 25% reoperation rate due to pseudarthrosis and no significant functional improvement.

### Total hip arthroplasty

Five systematic reviews assessed THA outcomes. Of these, three reviews focused exclusively on THA (37, 38, 39), while two included THA among other salvage procedures (35, 36). Across studies, THA was associated with improvements in pain, ROM, and activities of daily living (ADL). Complication rates ranged from 10 to 45% above those observed in the general population (most frequently prosthetic dislocation (1–29%), periprosthetic fractures (1.69–36%), and aseptic loosening (0.74–20%)). Revision rates ranged from 0 to 19%, and implant survivorship ranged from 85 to 100% at five years and from 73 to 86% at ten years. Kolman *et al.* (35) reported 93.8% pain relief and 70% functional improvement. Boldingh *et al.* (39) reported 91% pain relief, 45% improvement in sitting ability, and HO in 45% of cases.

### Avascular necrosis

Hesketh *et al.* (40) found AVN rates ranging from 0 to 46% after hip surgery. No clear links were found between AVN and age, subluxation severity, or surgery type. Variability in diagnostic methods and a lack of standardized definitions limit interpretation.

### Use of antifibrinolytics

Two systematic reviews evaluated antifibrinolytics. Rangasamy *et al.* (41) reviewed five studies (478 patients), three of which showed reduced total blood loss, and all reported no adverse effects. However, variability in dosages and a lack of standardized protocols limit the generalizability of the results. De Dios Domínguez *et al.* (42) conducted a meta-analysis of seven studies (943 patients), showing that TXA significantly reduced transfusion requirements and blood loss without notable side effects. Both reviews noted the need for more prospective studies to establish standardized protocols.

## Discussion

This umbrella review consolidates and critically appraises the current systematic evidence regarding screening, surveillance, and management strategies for hip instability in children with CP. It highlights both the breadth and limitations of the available literature and aims to guide future clinical practice and research directions.

### Radiological assessment

Radiological evaluations remain central to clinical decision-making. The reviewed studies support the validity and reliability of several radiographic parameters, including MP, AI, HSA, and femoral anteversion, which therefore appear to be standard methods for initial diagnosis and treatment (2, 19).

### Pain

Although pain is often cited as an indication for intervention, evidence linking hip displacement and pain remains inconsistent. Karkenny *et al.* (3) emphasize that variability in pain assessment methods, age ranges, and the absence of control groups weakens conclusions. Still, advanced displacement (MP > 50%) may be independently associated with greater pain in children with severe CP, highlighting the need for standardized pain assessment tools. While surveillance programs improved metrics such as MP, they did not consistently prevent hip pain or improve function or QoL. One study found that severe subluxation (MP 50–89%) poses the highest pain risk, whereas bilateral, painless dislocation may not require surgery unless pelvic obliquity, functional, and caregiving issues are present (43). Prioritizing functional outcomes and QoL through systematic data in longitudinal studies is essential for guiding care and setting realistic expectations with families.

### Hip surveillance

Multiple HS programs have been successfully implemented in different parts of the world (44, 45, 46). Two systematic reviews evaluated the effectiveness of HS in CP (8, 9), demonstrating a significant decrease in the need for reconstructive surgery and essentially eliminating the need for salvage procedures.

Gordon and Simkiss (8) found that HS is mainly needed for children with spastic quadriplegia and non-ambulatory diplegia by 30 months. MP and AI are key measures, although they require standardization; MP is more treatment-relevant and remains reliable beyond age 8. Wynter *et al.* (9) reported a linear link between GMFCS, femoral anteversion, NSA, and dislocation risk, while movement disorder type and topography were less predictive. Dislocation may occur early (7–11% of <2-year-olds with MP > 30%) and can progress after puberty if risk factors such as pelvic obliquity or scoliosis are present. Although ROM does not predict dislocation, it remains essential in surveillance (44, 45, 46). Wordie *et al.* (47) highlighted that an MP equal to or exceeding 46% represents a threshold beyond which spontaneous regression does not occur.

Pruszczynski *et al.* (48) evaluated hip displacement screening in 1,082 children with CP aged 18 or younger and found a linear correlation between MP and GMFCS. Displacement reached 30–33% by the ages of 3–5, with dislocation around 5–6 years. A higher risk was observed among children under 5 years old who were classified as GMFCS IV–V. While CP pattern risk was inconclusive, ataxic CP had a low risk, and dystonic and spastic types had a higher risk. The most significant risk was in GMFCS III–V between ages 2 and 8. Annual radiographs were advised for a 12% subluxation rate. Children classified as GMFCS V and under 5 years of age with an MP of 30–40% need imaging every 6 months. After age 8, children with GMFCS I–II require imaging only if MP > 39%, whereas those with GMFCS III–V should have radiographs every 2 years.

Based on our findings, HS should be initiated by age 2, with more frequent monitoring recommended for children classified as GMFCS IV–V, ideally beginning at diagnosis in younger children. Children at GMFCS I–II require less frequent follow-up, except Winters and Gage type IV hemiplegia, who remain at an increased risk. Children classified as GMFCS III also warrant closer surveillance due to delayed ambulation and femoral changes. Continued monitoring beyond skeletal maturity may be necessary for non-ambulatory patients with risk factors (8, 9).

### Postural and tone management

Despite systematic approaches, current studies on hip displacement prevention in CP are small, are retrospective, and lack standardized outcomes. Positioning, bracing, and weight-bearing strategies are commonly used, but evidence remains weak. Postural programs and sleep systems may slow hip migration, although most lack methodological rigor and randomized trials (10, 20, 21). Bracing alone shows no preventive effect, and standing frames improve bone density and spasticity but not hip stability (21, 22). Integrated strategies are promising but lack long-term data (23). An expert consensus review noted limited and conflicting evidence for bracing and BoNTA (9).

The effectiveness of BoNTA, SDR, ITB, and obturator nerve blocks in preventing hip displacement is uncertain due to low-quality evidence. BoNTA may relieve postoperative pain but does not prevent migration (25). SDR and ITB reduce spasticity, but their impact on hip stability is inconsistent (10, 24). Recent studies showed that hip subluxation occurred in 10.3% of patients post-SDR (49), and long-term follow-up showed no clear SDR benefit for the hip (50). In 49 patients who were treated with ITB, early pump implantation, MP > 31%, and ΔMP > 15% predicted surgery (51). High-quality, long-term studies are needed to clarify the role of these interventions.

### Orthopedic surgery

**Figure 2:**
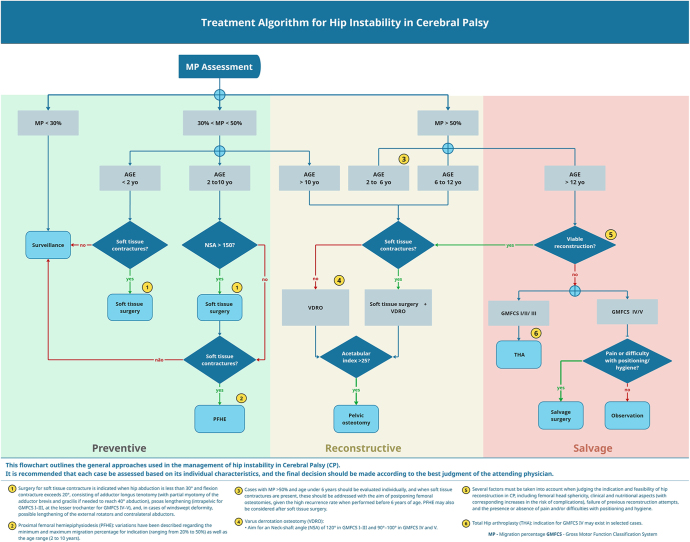
Treatment algorithm for hip instability in children with CP. Decision-making flowchart summarizing preventive, reconstructive, and salvage strategies for the management of hip instability in CP. The algorithm is based on MP, age, and Gross Motor Function Classification System (GMFCS), integrating evidence from recent systematic reviews. Preventive strategies include surveillance and soft-tissue surgery (adductor and psoas releases) or proximal femoral hemiepiphysiodesis (PFHE). Reconstructive procedures (varus derotation osteotomy (VDRO) and pelvic osteotomy) are indicated for MP > 40–50%, depending on acetabular dysplasia and femoral morphology. Salvage options (valgus osteotomy, FHR, or total hip arthroplasty (THA)) are considered when reconstruction is no longer viable. Each pathway emphasizes individualized assessment according to functional level, hip morphology, and symptomatology.

This systematic review underscores the complexity and evolving nature of strategies for managing hip displacement in children with CP, while highlighting the limited availability of high-level evidence to inform guideline development. Surgical treatment is typically divided into preventive procedures (such as soft-tissue releases and PFHE), reconstructive procedures (including VDRO and pelvic osteotomies), and salvage procedures (such as PFR, valgus osteotomy, and THA), with the choice guided by age, severity of contractures, MP, acetabular dysplasia, and femoral head deformity. To support clinical decision-making, these parameters were synthesized into a treatment algorithm (Fig. 2). Importantly, children may present at different stages of hip displacement depending on access to surveillance programs and specialist care; therefore, the proposed algorithm is intended to guide management based on observed clinical and radiographic findings, while decisions remain individualized, context-specific, and grounded in shared, family-centered decision-making.

Soft-tissue surgeries are considered interventions aimed at preventing the progression of hip instability and improving the range of motion. There is an indication that results are better for young children when subluxation is observed (MP > 30%) and hip abduction is limited (<30°) (12, 52). The MP < 30% at one year postoperatively was a good predictor of outcome (53).

This review indicates that the effectiveness of soft-tissue surgery alone remains unproven in preventing the progression of hip displacement (26). Nevertheless, procedures such as adductor and iliopsoas tenotomies may delay bony surgery or slow the progression of hip migration (54, 55). Postponing bone surgery until after age six appears to lower the risk of recurrence and complications. Given the limitations of soft-tissue surgery, PFHE has emerged as a minimally invasive option to promote femoral growth into varus and reduce subluxation. Lebe *et al.* published the only systematic review on the topic and reported improvements in MP (mean: 8.48%) and decreases in HSA (12.28°) and AI (3.41°) after at least 2 years of follow-up (28). Davids recommends PFHE for children aged 4–10 with MP between 25 and 50% (56). Portinaro *et al.* (29) support combining it with soft-tissue release when indicated. Complications included physis growing off screws in about 50% of cases and progressive subluxation requiring osteotomies in 5–21%. Screw positioning, patient age, and use of arthrography control were essential factors. The screw should be placed in the medial portion of the proximal femoral physis to guide the growth to decrease the NSA and hip displacement. Regarding age, all the articles in the study report an age range of 4–12 years, primarily due to the growth potential of the proximal femoral physis. As for arthrography, it is important in younger patients in whom the femoral head is still largely cartilaginous. The study’s main limitation was its reliance on small, short-term cohort studies. Further research is needed to refine indications, timing, and implant choice. Howard (12) recommended PFHE with soft-tissue release for HSA > 155° and MP 30–50% in children aged 4–10. While PFHE addresses proximal femur valgus and mild acetabular dysplasia, it does not correct increased femoral anteversion. Surgical decision-making for early hip displacement in children with CP remains a balance between risks and benefits. Less invasive procedures, such as PFHE, have lower initial complication rates but often require additional surgeries. In contrast, VDRO carries a higher surgical risk but may achieve definitive correction in a single intervention. Family preferences and priorities should play a central role in this decision.

The literature on reconstructive procedures is limited by mostly observational designs, few prospective trials, small samples, and patient heterogeneity. Reconstructive surgery is generally indicated when MP exceeds 40% without severe femoral head loss of sphericity, which may indicate chondrolysis and exposure of subchondral bone (11). The choice between femoral, pelvic, or combined osteotomies depends on subluxation severity and acetabular dysplasia. According to Bouwhuis *et al.* (26), combined surgeries, VDRO and pelvic osteotomy, yielded superior results, with a higher percentage of hips achieving MP below 33% (mean follow-up: 3.6–19.1 years) and significantly fewer cases of resubluxation and redislocation compared to VDRO alone.

Although there is no established consensus regarding the indications for pelvic osteotomy, Park *et al.* (57) have proposed its use in cases of severely subluxated or dislocated hips, as well as instances where VDRO successfully reduces the femoral head but the acetabulum is dysplastic. An AI > 25° typically supports pelvic osteotomy. In long-standing dislocations, acetabular dysplasia can be significant. Intraoperative arthrography can help in the decision to perform a pelvic osteotomy in cases where femoral head coverage is not sufficient after VDRO (12). Among the various techniques, the Dega osteotomy is preferred for its enhanced posterior and lateral coverage (12).

According to Davids (58), an open reduction to remove soft-tissue obstacles is only indicated in cases of complete dislocation where the femoral head has migrated proximally, and there is no contact with the acetabulum (i.e., MP > 100%).

This review found no clear consensus on unilateral versus bilateral surgery for unilateral hip instability. However, bilateral procedures are generally preferred, as they reduce the risk of contralateral displacement, correct bilateral femoral anteversion and valgus, and preserve symmetry (59). Noonan *et al.* (60) reported that only 26% of hips remained stable after unilateral surgery. In non-ambulatory children, predictors of failure include the absence of contralateral soft-tissue release, reversal of pelvic obliquity, MP > 25%, and age <8 years (12).

This review found no formal consensus regarding the optimal degree of varus correction in VDRO. Presedo *et al.* (61) recommended targeting an NSA of 120° in children classified as GMFCS I–III who have potential for long-term ambulation and around 100° with 10°–20° of anteversion in those classified as GMFCS IV–V. In more severely affected patients, the VDRO technique should also involve removal of the lesser trochanter and iliopsoas insertion (62).

Studies in this review rarely specified the type of abnormal tone. Although spastic children commonly develop hip instability, hypotonic or dystonic hips are discussed mainly for pathophysiological rather than outcome reasons. However, surgical planning must consider tone patterns. In spastic hips, displacement results from adductor, iliopsoas, and hamstring contractures (53, 62), with early management including soft-tissue release or PFHE (54) and later requiring stable reconstruction, such as VDRO with or without pelvic osteotomy (63). Hip instability in hypotonia results from poor containment and abductor weakness. Soft-tissue procedures are less effective here, and durable bony containment through hemiepiphysiodesis or osteotomy is preferred (32, 33). Dystonic hips differ, as fluctuating involuntary muscle activity creates unpredictable forces, often requiring adjunctive tone management (e.g., intrathecal baclofen and deep brain stimulation) alongside reconstruction (64, 65). Further studies are needed to define optimal strategies for each tone type.

There are no established guidelines on whether to address hip instability or scoliosis first when both are present. However, Helenius *et al.* (66) suggested prioritizing spinal fusion in cases of significant spinal curves or pelvic obliquity, especially when curves impair breathing, as leveling the pelvis can improve overall alignment and function. The relationship between scoliosis and hip instability in CP has been reported, particularly among non-ambulatory children and those with higher GMFCS levels. Hip displacement, pelvic obliquity, and scoliosis frequently co-occur, with evidence indicating that hip displacement often precedes scoliosis development. Longitudinal studies demonstrate that severe unilateral hip displacement is associated with scoliosis convexity opposite to the displaced hip, suggesting that hip displacement and PO are key contributors to scoliosis pathogenesis (67, 68). The prevalence of both deformities increases with higher GMFCS levels, older age, and severe postural asymmetries, which also raise the risk of windswept hips, contractures, pain, and functional limitations (69). Predictors of severe scoliosis include poor motor function, spasticity, and previous hip surgery, with the latter identified as a strong independent risk factor (70). Hip displacement further contributes to scoliosis progression, especially in children with early-onset scoliosis or an elevated Cobb angle (71). Surgical correction of scoliosis, including posterior spinal fusion, does not significantly alter hip displacement progression, even when PO is addressed, indicating the complex, multifactorial nature of these deformities (72).

High complication rates after reconstructive hip surgery, including death, have been reported, particularly in complex patients with gastrostomies, epilepsy, non-verbal communication, and tracheostomies, where overall complication rates can rise to 68% (73). According to the literature, reinterventions ranged from 13.9 to 37% (11, 33). Recurrence of hip dislocation or subluxation after surgery in children with CP is multifactorial. Radiographic factors are the strongest predictors: a preoperative MP > 70% and AI > 25° increase the risk of recurrence, while achieving a postoperative MP < 27.5% and AI < 23.1° improves outcomes. Residual subluxation or acetabular dysplasia raises failure rates. For soft-tissue-only procedures, a post-op MP < 32% is associated with better outcomes, and bony reconstruction should be considered when pre-op MP > 44% (74, 75). A preoperative AI < 34° predicts a better prognosis after soft-tissue release (76). Age and timing are also important. Reconstructive surgeries performed before the age of 6 years are associated with higher recurrence rates, especially in severe displacements, whereas older age is associated with better outcomes in less severe hips (74, 77). A higher pre-op MP and displacement velocity increase the risk of redisplacement and reoperation after both soft-tissue and bony procedures (4). Surgical technique influences results. Adding a pelvic osteotomy to femoral varus derotation osteotomy (VDRO) improves outcomes in severe subluxations (MP > 50%), while capsulorrhaphy shows no benefit and may decrease success (77). Correcting acetabular dysplasia during reconstruction is protective (74, 77). Functional level is a strong predictor: children with GMFCS IV–V have faster postoperative progression of MP and a higher recurrence rate, requiring close follow-up (78, 79). Pelvic obliquity and male sex further increased risk in non-ambulatory patients after triradiate cartilage closure (79).

AVN is a painful and troublesome complication, although the true incidence may be underestimated due to inconsistent diagnostic criteria, with a reported rate of 7.5% in this review (40).

Control of blood loss is a critical aspect of hip reconstruction surgeries. TXA appears to reduce the need for transfusions and minimize blood loss, with no significant adverse effects reported.

### Salvage procedures

Salvage procedures are used for painful, irreducible hips when reconstruction fails and there is severe arthritis. Goals include pain relief, improved mobility, better hygiene, and improved QoL. Options include THA, FHR, valgus osteotomy, HA, and interposition arthroplasty, with selection based on age, GMFCS level, deformity, pain, and function. No technique is clearly superior (12), and most evidence is level IV, with limited standardized outcomes.

The THA is usually recommended for ambulatory patients with GMFCS levels I–III to restore joint function. Pain relief occurs in up to 93.3% of cases, and functional improvements are seen in up to 80% (35, 39). Implant survival rates range from 85 to 100% at five years and 73–86% at ten years (36, 39). Common complications include prosthetic dislocation (1–29%) (36, 38), periprosthetic fractures (1.69–36%) (41), aseptic loosening (0.74–20%) (39), and HO (up to 45%). Revision rates range from 0 to 26.6%, mainly due to loosening (38), with ten-year revision rates of 5–7% in the general population but potentially over 25% in CP patients.

For non-ambulatory patients (GMFCS IV–V), FHR and valgus osteotomy (VO) achieve success rates of 89–90.4% (34, 35). The FHR, alone or combined with VO (McHale technique), is commonly used for chronic dislocations and non-reconstructable hips (36). Although FHR below the lesser trochanter (Castle–Schneider procedure) avoids implant-related risks, complications such as proximal femoral migration (17–28%) and heterotopic ossification (41–62%) remain frequent (34). Postoperative pain often resolves within 3–15 months, and traction-free protocols are favored to promote early mobilization and reduce morbidity. The VO is generally well tolerated with low failure rates, and interpositional arthroplasty shows promising results, especially with pelvic stabilization (38). The HA has high complication rates, including up to 25% pseudarthrosis and severe stiffness, with adverse events often exceeding procedures performed (35, 36). Due to inferior outcomes and higher morbidity, HA is generally not recommended.

### Limitations of the study

While this umbrella review offers a broad synthesis of the literature, several limitations must be acknowledged. Many of the included systematic reviews were rated as critically low to low quality according to AMSTAR 2 criteria, and heterogeneity in populations, interventions, and outcome measures limits direct comparisons. Furthermore, studies often lacked standardized definitions, particularly regarding pain and functional outcomes.

### Future research

Future research on hip instability in children with CP should include high-quality randomized controlled trials evaluating non-surgical interventions, such as physical therapy, orthotics, and pharmacologic options, as well as tone management measures, to define their roles in early management better. Standardized outcome reporting is essential, with a focus on meaningful measures, such as pain, QoL, and caregiver burden, which are often underreported despite their clinical relevance. Long-term comparative studies of reconstructive and salvage surgical procedures are also needed to recommend treatment options, particularly regarding durability, function, and patient satisfaction. In addition, the use of pediatric-specific evaluation tools and radiographic classification systems would enhance consistency in diagnosis, treatment planning, and outcome assessment across clinical and research settings.

## Conclusion

This umbrella review analyzed systematic reviews on hip instability in children with CP, covering screening, surveillance, and treatment strategies. While some evidence supports early surveillance and interventions, such as combined osteotomies, most areas lack robust data due to low methodological quality, limited long-term follow-up, and variability in interventions. The findings emphasize the need for individualized treatment based on GMFCS level, age, radiographic findings, and pain, as well as the importance of high-quality studies to guide future clinical practice.

## Supplementary materials









## ICMJE Statement of Interest

The authors declare that no conflict of interest could be perceived as prejudicing the impartiality of the research.

## Funding Statement

This research did not receive any specific grant from any funding agency in the public, commercial, or not-for-profit sector.
