# Supplementary material for: The approach to hip instability in children with cerebral palsy: an umbrella review
Source: EFORT Open Rev. 2026 Mar 2;11(3):208–23. doi: 10.1530/EOR-2025-0114 (PMC12974736; doi:10.1530/EOR-2025-0114)
Supplement: Supplementary file 1 [file supplementary_materials.pdf]

Appendix I: Search strategy

Keywords

Terms connected by **OR**	**AND**	Terms connected by **OR**
"Cerebral Palsy" [MeSH terms]		"hip" [MeSH terms]

Filters

- Publication date: since 2004 (last 20 years)
- Type of study: Systematic review

MEDLINE via PubMed

Search conducted on 7/31/2024

Results retrieved (English terms) 57

("cerebral palsy"[MeSH Terms] OR ("cerebral"[All Fields] AND "palsy"[All Fields]) OR "cerebral palsy"[All Fields]) AND ("hip"[MeSH Terms] OR "hip"[All Fields]) AND ((systematicreview[Filter]) AND (2004:2024[pdat]))

Web of Science (includes Scielo)

Search conducted on 7/31/2024

Results retrieved (139)

(ALL=("cerebral palsy" AND hip)) AND DT==("REVIEW") AND FPY=2004-2024

BVS

Search conducted on 7/31/2024

Results retrieved 69

("cerebral palsy") AND (hip) AND (collection:("06-national/BR" OR "05-specialized") OR db:("LILACS" OR "MEDLINE")) AND (type_of_study:("systematic_reviews")) AND (year_cluster:[2004 TO 2024])

Scopus

Search date: 7/31/2024

Results retrieved 235

TITLE-ABS-KEY-AUTH ("cerebral palsy") AND TITLE-ABS-KEY-AUTH (hip) AND DOCTYPE (re) AND PUBYEAR > 2003

PEDro

Results retrieved 14

Date searched 7/31/24

Abstract & Title:	"cerebral palsy" AND hip
Therapy:	
Problem:	
Body Part:	
Subdiscipline:	
Topic:	
Method:	systematic review
Author/Association:	
Title Only:	
Source:	
Published Since:	2004 [YYYY]
New records added since:	[DD/MM/YYYY]
Score of at least:	[/10]
Return:	50 records at a time
When Searching:	Match all search terms (AND)
	Match any search term (OR)

Start Search

CINAHL

Results retrieved 131

Date searched: 7/31/24

	Search ID#	Search Terms	Search Options	Actions
	S1	"cerebral palsy" AND hip	**Limiters** - Publication Date: 20040101-20241231; Publication Type: Systematic Review<br>**Expanders** - Apply related words; Also search within the full text of the articles; Apply equivalent subjects<br>**Search modes** - Find all my search terms	View Results (131) View Details Edit

EMBASE

Results retrieved 112

Search date: 7/31/24

('cerebral palsy'/exp OR 'cerebral palsy') AND ('hip'/exp OR hip) AND 'systematic review'/de AND (2004:py OR 2005:py OR 2006:py OR 2007:py OR 2008:py OR 2009:py OR 2010:py OR 2011:py OR 2012:py OR 2013:py OR 2014:py OR 2015:py OR 2016:py OR 2017:py OR 2018:py OR 2019:py OR 2020:py OR 2021:py OR 2022:py OR 2023:py OR 2024:py)

Cochrane Library

- The cochrane library is indexed in MEDLINE, WoS, EMBASE, CINAHL and Scopus

Total results 757
