# Supplementary material for: The approach to hip instability in children with cerebral palsy: an umbrella review
Source: EFORT Open Rev. 2026 Mar 2;11(3):208–23. doi: 10.1530/EOR-2025-0114 (PMC12974736; doi:10.1530/EOR-2025-0114)
Supplement: Supplementary file 2 [file supplementary_table_1.pdf]

Supplementary Table 1. Orthopedic surgery studies.

Author, Year	No of Studies<br>Level of Evidence	Population/GMFCS	Age at surgery/ follow-up	Results/ Complications	Authors'conclusion
Agarwal et al., 2019 [33]	38 resubluxation/reoperation Level III: 14.<br>MP 24 studies<br>Levels:<br>I: 1<br>II: 1,<br>III: 10<br>IV: 12	729 hips resubluxation/reoperation.<br>MP 1492 hips<br>II, III, IV, V (only six papers reported GMFCS levels)	Median: 7.4 y (1-17).<br>Follow up: 0.4-16.7 y.<br>Three studies did not stipulate a minimum follow-up time.	The odds of RsR were significantly lower for FO + PO compared with FO (OR = 0.49; 95% CI 0.25 to 0.98).<br>The odds of RsR were significantly lower for FO compared with STS (OR = 0.20; 95% CI 0.07 to 0.61).<br>The odds of RdR were not statistically different for PO versus VDRO (OR = 2.27; 95% CI 0.37 to 13.88). MP: VDRO + PO improved 60%. Isolated PO improved 45%. Isolated FO improved 33%, STS 10% and negligible improvement with BoNT-A	Did not support the use of BoNT-A in treatment of hip subluxation.<br>STS:moderate improvement in MP; reoperation/resubluxation 50% to 77%; Combined PO + FO and femoral osteotomies provided the greatest improvement in MP and the lowest odds of subsequent resubluxation/redislocation/reoperation.
Bouwhuis et al., 2015 [26]	10<br>STS:1<br>BS:9<br>level: IV	STS: 38 pts/ 70 hips<br>BS:189 pts/289 hips IV, V	STS: mean 5.5 y (2.4 to 9.7)<br>BS: mean varies from 8 to 10 y (2 to 22 y)<br>follow-up:<br>STS: 7.4 y (1.7–19.2)<br>BS: mean 3.6 to 19.1 y	table at follow-up:<br>STS-MP<50%: 87% of the hips. MP>50%: 43% of the hips<br>BS-MP pre-op 61 to 100%, MP post-op 6 to 29%<br>MP < 33% post-op: 53–75% for VDRO alone and VDRO + PO: >=85%<br>Complications BS (pts): femur fracture (12), pressure ulcer (7), avascular necrosis (3), limb length discrepancy (2), superficial infection (1), osteoarthritis (8), HO (8)	STS indicated in specific cases, alone, not sufficient to treat hip subluxation or dislocation, particularly in children with greater CP severity. If soft tissue surgery fails in preventing or treating hip displacement: indication for VDRO with or without PO in children with severe CP. Older patients with severe acetabular dysplasia: VDRO and PO.
El-Sobky et al., 2018 [32]	36<br>Level II: 1<br>Level III: 35	1,771 pts/2,568 hips (293 had no GMFCS reported)<br>III (145),IV (367),V(966)	Mean 3.9 to 14.6 y<br>Mean follow-up: 2 to 19 y	VDRO + PO: 1374 hips (53.5%)<br>MP pre op: 55-100% (follow-up>6 y) post-op: 4,3 - 11% (follow-up 7.9-25%)<br>Isolated VDRO: 1173 hips (45.6%)<br>Isolated PO: 21 hips (0.8%)<br>All osteotomies pts had concomitantly STS (adductor musculature w/ or w/o iliopsoas tenotomy, or part of a SEMLS). Most common PO used: Dega and Pemberton	Fair evidence for STS and combined pelvifemoral reconstruction in the management of hip migration in nonambulatory and minimally ambulatory CP children in the short and long term. There is limited evidence available that would support the use of soft tissue and isolated femoral reconstruction.<br>The retrospective nature of articles showed a significant number of confounding variables that

				periacetabular. Less common: Salter, peri-ilial, shelf acetabular augmentation, Albee and Sutherland double osteotomy and triple osteotomy<br>Complications: pain, AVN, reoperation or revision, redislocation, coxa vara, graft dislocation, osteoarthritis, infection, hardware failure, sitting problems, deterioration of motor functions, HO, recurrent contraction, pathologic fracture and decubitus ulcer.	limited the validity and generalizability of conclusions.
Lebe et al., 2022 [28]	4<br>level: IV	93 pts/<br>178 hips<br><br>I(7), II(10), III(24), IV(31), V(21)	mean: 6,2 - 8 y (4 to12 y)<br>follow-up: 45 - 50 mo (2 to 8 y)	Mean change nges:MP: 8.48% (95% CI 3.81–13.14)<br>HSA: 12.28 (95% CI 11.17–13.39)<br>AI: 3.41 (95% CI 0.72–6.10).<br>Physeal growing off of the screw: 43%<br>Progressive hip subluxation: 5% to 21%	Guided growth of the proximal femur, with concomitant STS if necessary (adductors, psoas and hamstrings), is an effective and predictable method to treat CP patients with ‘hips at risk’, and the overall complication rate is low. Further work is required to identify best candidates, surgical timing and choice of technique and implant.

Legend: Nº: number; pts: patients, y: years; GMFCS: Gross Motor Function Classification System, VDRO: femoral varus-derotational osteotomy, PO: pelvic osteotomy, AVN: avascular necrosis, MP: Migration Percentage , HSA: head-shaft angle, AI: acetabular index, TXA: tranexamic acid, SEMLS:single event multiple level surgeries, OR: odds ratio, CI: coefficient intervals, TXA: tranexamic acid, TBL: total blood loss, EBL: estimated blood loss, Hb: hemoglobin, Hct: hematocrit., EACA:ε-aminocaproic acid, BS: bone surgeries, STS: soft tissue surgeries, SEMLS: single event multilevel surgery, HO: heterotopic ossification, FO: femoral osteotomy; RsR: Ressubluxation/Reoperation; RdR: Redislocation/Reoperation.
