# Supplementary material for: The approach to hip instability in children with cerebral palsy: an umbrella review
Source: EFORT Open Rev. 2026 Mar 2;11(3):208–23. doi: 10.1530/EOR-2025-0114 (PMC12974736; doi:10.1530/EOR-2025-0114)
Supplement: Supplementary file 3 [file supplementary_table_2.pdf]

Supplementary Table 2. Hip Salvage Surgery studies.

Author , Year	Nº of Studies/ Level of Evidence	Population/GMFCS	Age at surgery / Follow-up	Results / Complications	Authors'conclusion
Boldingh et al., 2013 [36]	8<br>FHR: 6 (McCarthy)<br>THA: 1<br>Arthrodesis: 1<br>Level : IV.	100 patients<br>FHR: 82<br>Castle: 22<br>McCarthy : 60<br>THA: 11<br>Arthrodesis: 7<br>GMFCS: IV, V	3 - 45 y:<br>THA: 11- 20<br>FHR: 3 - 45<br>Arthrodesis:10-17.<br><br>Follow-up: 1 y<br>FHR: 1 - 20<br>McCarthy: 1- 20.8<br>Castle: 6 months – 7 years.<br>THA: 2 – 6.5 years.<br>Arthrodesis: 2 – 7 years.	FHR (Pain relief: McCarthy: 53 - 77%, Castle: 90 100%; Improvement in sitting: all; Improvement nursing / problems/care: McCarthy: 92%, Castle: 100%). THA (Pain relief : 91%; Improvement in sitting: 45%; Improvement Nursing problems/care: all). Arthrodesis (Pain relief: all; Improvement in sitting: 71,42%; Improvement Nursing: problems/care: all)<br><br>Complications: FHR (HO (41% - 62%): McCarthy: 41-62%, Castle: 0-57%; Proximal migration (17-28%): McCarthy: 41-62%, Castle: 0-57%)). Other: pressure ulcers and pain (22 cases): (McCarthy: 18 cases (pressure ulcers) and postoperative pain (20 cases). THA: Dislocation of the prosthesis (29%), femoral fractures (36%) and HO (45%). Arthrodesis: Pseudartrose (42%), femur fracture (one patient))	Favorable effect on pain and sitting/nursing problems.No clearly favor one procedure over the others. Castle procedure has best results and fewer complications, with good outcomes. Hip arthrodesis no evidence of pain relief and improvement in ADLs
Kolman et al., 2016 [35]	28<br>DP: 11<br>FHR: 1<br>VO: 2<br>THA: 6<br>SPI: No<br>Arthrodesis:1<br>NP: Not depicted<br>Level: IV.	722 hips<br>GMFCS V: one THA (DP/NP);<br>GMFCS IV: FHR=369(51.1%); VO: 160 (22.2%); THA: 139 (19.3%); SPI: 32 (4.4%); arthrodesis: 22, 3%<br>DP (172 patients / 200 Hips): FHR: 28/42; VO: 27/33; THA: 113/121; SPI: No; Arthrodesis: 4/4<br>NP: 486 procedures:<br>FHR: 313; VO: 126; THA: 17; SPI: 14; Arthrodesis: 16.<br>Follow-up: Not depicted	Age at surgery: Not depicted<br><br>Follow-up: > 1 y	NP (OR/95%): Pain relief (FHR: 90,4%/7.3/2.2-24.8; VO: 88,4%/5.9/1.6-22.8; THA: 93,8%/11.7/1.1-297.5; SPI: 90,9%/no/no; Arthrodesis: 56,3%/no/no). OR/CI: FHR/VO/THA> Arthrodesis.<br>DP: Pain relief: 93.3%.<br><br>Complications: All techniques: 24% – 100%; DP (PXC): FHR: 42/16 (PMF; HO (most common); VO:34/16 (complications mainly related to implants); THA: 121/52 (revision (14.7%)<br>NP (PXC): FHR: 77/313 (24%): PMF: 7.7%; HO: 3.2%; VO: 44/126 (33.3); symptomatic implants:13.5%; infection:5.6%; THA: 6/17 (35.3%) - revision: 17,6%; SPI: 4/14 (28.6%) - femur fracture: 14,3%.; Arthrodesis: 17/16 (106.3%) - revision for nonunion: 25%.<br>x2 / Fisher: FHR ($P < 0.001$), VO ($P < 0.001$), and THA ($P < 0.001$).	FHR and THA demonstrated favorable outcomes for pain relief and improvement in ADLs; HA indicated for non-ambulatory patients for pain relief, due to the high complication rates

				DP/NP: Arthrodesis: >100%.(pseudarthrosis: 25%).	
Souza et al., 2015 [34]	10 Retrospective Cohort:<br>175 patients<br>Arthroplasty: 1<br>Arthrodesis: 1<br>McHale: 1<br>Schanz: 1<br>PRF(Castle): 6<br>Level: IV	Arthroplasty (THA +SPI): 11 (NP/ tetraparetic/skeletally mature);<br>Arthrodesis: 14 (diplegic: 4; quadriplegic: 8; mixed pattern*: 2);<br>DP: 4 (household: 1; sitters: 3; permanently bedridden: 7).<br>Castle: 94 (tetraparetic:+ / GMFCS: V**).<br>McHale: 21<br>Schanz: 35	Mean: 14.7 y (10.1 - 17.6):<br>Arthroplasty: 17<br>Arthrodesis: 15.4<br>Castle: 17.6<br>McHale: 10.1<br>Schanz: 14.72<br><br>Follow-up:<br>Mean(mo): Arthroplasty: 57<br>Arthrodesis: 63<br>Castle: 53.9<br>McHale: 54.5<br>Schanz: 98<br>Overall Mean: 65	THA: Improved pain and ADL; Schanz and McHale:<br>Pain relief and ADL: > 80%. -Persistent pain: 7.3% - 15.3%.<br>Castle and Schneider: -improvement pain, ROM: all studies improvement ADLs: 62%-100%<br>Castle vs. McHale: Castle > McHale<br><br>Complications: THA: persistent difficulties in hygiene and hip pain: 1; Schanz and McHale: HO,implant failure, postoperative infection, and persistent pain.; Castle and Schneider: femoral stump ascension, pain recurrence or lack of improvement, and HO.	THA is indicated in older, skeletally mature, ambulatory and better functional level patients; success linked to the severity of CP. Hip salvage surgeries need individualized evaluation<br>No single superior surgical technique; rather, different techniques have specific indications.

Nº: number/ *mixed pattern refers to spastic/dyskinetic/ **Reported in only one study/PC: Cerebral Palsy. GMFCS: Gross Motor Function Classification System. THA: Total Hip Arthroplasty. FHR: Proximal Femur Resection. SPI: Hip Interposition Arthroplasty. VO: Subtrochanteric Valgus Osteotomy. HO: heterotopic ossification. ADLs: activities of daily living. N: Non Ambulatory patients. D: Ambulatory patients. DP/NP: Nonambulators and Ambulatory patients. PXC: Procedures/Complications. PMF: proximal migration of the fêmur.
