# Supplementary material for: The approach to hip instability in children with cerebral palsy: an umbrella review
Source: EFORT Open Rev. 2026 Mar 2;11(3):208–23. doi: 10.1530/EOR-2025-0114 (PMC12974736; doi:10.1530/EOR-2025-0114)
Supplement: Supplementary file 4 [file supplementary_table_3.pdf]

Supplementary Table 3. Total Hip Arthroplasty studies.

Author, Year	Nº of Studies/ Level of Evidence	Population/GMFCS	Age at surgery / Follow-up	Results / Complications	Authors' conclusion
Adams et al., 2020 [39]	9<br>Level: Not depicted	Hips: 609<br>Patients: 170 (incomplete report)<br>GMFCS: I(3); II(18), III(12); IV(6); V(0)	Minimum mean age: 19.2 y (13.5–31.6)<br>Maximum mean age: 54 y (43 - 61)<br>Mean follow-up range: 3.3-10 y	Pain relief: 87%<br>Functional: 1 study: 79%; Remaining studies: improvements in ADLs, sitting ability, transfers and ROM; Revision: 0% -> 15%<br>Implant survival (4 studies): 2 y: 92 – 95%; 5 y: 93.6%; 10 y: > 80% (81 - 95%); 15 y: 81%.<br>Complications (up to 26%): most common: recurrent prosthetic dislocation, periprosthetic hip fracture; Other: aseptic loosening, periprosthetic joint infections, complications of trochanteric osteotomy (hardware failure, nonunion, trochanteric bursitis, malunion), HO, wound dehiscence, and DVT.	Primary THA, alone or with soft tissue and/or osseous procedures, generally provides effective pain relief, improves ADLs, enhances sitting and transfer ability, and restores functional gait.
Costa et al., 2023 [37]	21<br>Level II: xx studies.	4.886 patients. Hips: 5 - 2.062.<br><br>GMFCS: I (10); II (45); III (44); IV (46); V (78).	Mean: 5 - 56,3 y<br>Follow-up:<br>Range: 3 mo - 10.5 y.	Pain relief: 15 studies (81% - >95%);<br>Functional improvement (independence in activities: sitting, ambulation, transferring): 11 studies; Improvement ROM: 3 studies (>85%); Revision rate (implant): 8.8% (0% - 19%) (Hip dislocation, aseptic loosening); Survival rates: 5 years: 85% to 100%; 10 years: 73% to 86%; 15 years: 81%.; Most common: anterior e posterolateral approach, uncemented prosthesis.<br>Complications: Prosthesis dislocation: 7.5% (0% - 28%), Periprosthetic fracture: 5,6% (0% - 21%); HO: 4.2% (0% - 37.8%); Loosening of the acetabular/femoral component: 3.7% (0% - 15.4%); Infection: 2.1% (0% - 16.6%).	THA is a good indication for patients presenting with joint deformities, hip dislocation, and degenerative arthritis.
Larrague et al., 2022 [38]	15<br>Level III: 3 studies.<br>Level IV: 12 studies.	603 (reported in 12 studies).<br>Hips: 2732<br>GMFCS (Reported in six studies): I(10); II(45), III(44); IV(46); V(78)<br>Classification in other studies: quadriplegia; combination of hemiplegia, diplegia,	Mean:17–79 y (11 - >85)<br>Follow-up: Mean: 3 - 10.1 y(4 mo - 16.8 y)	Favorable outcomes, effective pain relief, improvement of ADLs, and gait. Revision rate (0-26.6%); aseptic component loosening (1.5–13.3%); deep infection (1.6–2.56%); periprosthetic fracture (1.6–1.7%); dislocation (1–10.5%).<br>Implant survivorship: 84% (81–86%); Mean survival at 10 years: 85%<br>Complications (10 a 45%): dislocation: 1–20%; acetabular or femoral component loosening: 0.74–20%; periprosthetic femoral fracture: 1.69–10%; infection: 0.3–16.6%; HO: 2.5–53%; other: unremitting pain (1.2–12%), bursitis (8.4–20%), pressure ulcer (6.2–8.3%) and urinary tract problems (0.2–	THA is beneficial for painful osteoarthritis or a chronically dislocated hip. Controversy about the best type of implant fixation (i.e. cemented versus cementless) and the most appropriate acetabular insert (i.e. constrained versus unconstrained).

	triplegia, and quadriplegia.	3.48%)	

PC: Cerebral Palsy. GMFCS: Gross Motor Function Classification System. THA: Total Hip Arthroplasty. FHR: Proximal Femur Resection. SPI: Hip Interposition Arthroplasty. VO: Subtrochanteric Valgus Osteotomy. HO: heterotopic ossification. ADLs: activities of daily living. N: Non-ambulatory patients. D: Ambulatory patients. DP/NP: No ambulators and Ambulatory patients. PXC: Procedures/Complications. PMF: proximal migration of the femur.
